# Integrating Geometric Data into Topology Optimization via Neural Style Transfer

**DOI:** 10.3390/ma14164551

**Published:** 2021-08-13

**Authors:** Praveen S. Vulimiri, Hao Deng, Florian Dugast, Xiaoli Zhang, Albert C. To

**Affiliations:** 1 Department of Mechanical Engineering & Materials Science, University of Pittsburgh, Pittsburgh, PA 15260, USA; praveen.vulimiri@pitt.edu (P.S.V.); had50@pitt.edu (H.D.); fld5@pitt.edu (F.D.); 2 Department of Mechanical Engineering, Colorado School of Mines, Golden, CO 80401, USA; xlzhang@mines.edu

**Keywords:** topology optimization, neural network, neural style transfer, additive manufacturing

## Abstract

This research proposes a novel topology optimization method using neural style transfer to simultaneously optimize both structural performance for a given loading condition and geometric similarity for a reference design. For the neural style transfer, the convolutional layers of a pre-trained neural network extract and quantify characteristic features from the reference and input designs for optimization. The optimization analysis is evaluated as a single weighted objective function with the ability for the user to control the influence of the neural style transfer with the structural performance. As seen in architecture and consumer-facing products, the visual appeal of a design contributes to its overall value along with mechanical performance metrics. Using this method, a designer allows the tool to find the ideal compromise of these metrics. Three case studies are included to demonstrate the capabilities of this method with various loading conditions and reference designs. The structural performances of the novel designs are within 10% of the baseline without geometric reference, and the designs incorporate features in the given reference such as member size or meshed features. The performance of the proposed optimizer is compared against other optimizers without the geometric similarity constraint.

## 1. Introduction

With recent advances in additive manufacturing, it is now more feasible to fabricate complex designs generated by topology optimization. Topology optimization is a mathematical analysis of a design space, optimizing the material distribution to improve performance for a given metric (i.e., compliance, stress, etc.). Beginning with the work by Bendsoe and Kikuchi [1], the field has grown to include new approaches such as solid isotropic material with penalization (SIMP), level set (LS), and bi-directional evolutionary structural optimization (BESO) [2,3] and various design problems such as static, dynamic, thermo-elastic, and manufacturability [4,5]. These advances all improve the functional use of the optimized design. In fields where aesthetics add to the use of a design, such as architecture or art, the visual features of the design also contribute to the value. Such features have yet to be considered in detail for topology optimization.

The task of modifying the topology optimized design for geometric style then becomes the designer’s goal. Depending on the application, the visual appeal of a design also contributes to the overall performance, such as the air intake vents for a vehicle [6]. As a designer iterates between possible solutions, the final design may greatly deviate from the optimized result and decrease performance to satisfy the desired aesthetics. Rather than have a designer post-process the topology optimized result for form, it would be suitable for the analysis to simultaneously optimize for both performance and geometric features.

For example, texture synthesis integrated with topology optimization has been researched previously to perform a structural performance and geometric style optimization simultaneously [7,8]. These works sample regions from a given example texture and the optimized design to compare the appearance energies. The optimizer then minimizes the appearance energy subject to compliance and volume constraints. As these works are based on direct region similarity, there are still issues with applying the desired design concepts to the new design. The input must have patterns of similar size to the search region and have stochastic features. Otherwise, the final design would have many disconnected members not contributing to the performance. A full review of stylized design and fabrication can be found in Bickel et al. [9].

Although Wu et al. does not use by-example texture synthesis methods, the work constrains the amount of material in local regions of the design to achieve trabecular lattice structures [10]. A local mass constraint in these regions rather than using an example input creates these structures [11,12]. The work presents an excellent example of biomimicry for topology optimization. However, it is limited to only this lattice structure.

As an alternative to single design outputs, generative design is a recent design process to produce multiple outputs rather than a single result to satisfy a given condition. Genetic algorithms have been used to produce many different designs [13]. The genetic algorithm tests many samples within the design space and iterates on the highest performing samples, adjusting the variables according to their performance until an optimal sample is found. Autodesk has invested much in generative design research with multi-objective genetic algorithms [14]. However, producing these many designs requires large computational resources to achieve a variety of designs from which a designer should choose the best candidate.

Generative adversarial networks (GAN) have been shown to produce a wide variety of designs [15]. A GAN consists of two neural networks, a generator, and a discriminator, competing with each other. The generator attempts to design new structures similar to a database for a discriminator to discern which are from the database and which are from the generator. Following training, the generator produces structures indistinguishable from those in the database. One such work uses generative design for topology optimization [16]. The examples from the work show a wide variety of designs; however, the training database required hundreds of designs suited for the chosen design problem. This would be infeasible for a generic design tool, as thousands of designs would be needed to cover all design problems. Other examples of machine learning for topology optimization have focused on improving the computational time necessary to complete the analysis through training convolutional neural networks (CNN) [17,18,19,20]. These also require large amounts of training data to solve a specific design problem.

In this work, a pre-trained image classifier CNN provides differentiable extraction of geometric features in a design and reference. The geometric features and performance of a design are optimized simultaneously using a weighted objective loss function including neural style transfer [21] of a user-defined reference and topology optimization constraints including compliance, mass, and standard deviation. The neural style transfer uses the convolutional layer activations of a previously trained CNN to quantify the style of input. From the CNN’s previous training, the convolutional layers are accurate filters to extract the characteristics of the input. The calculation is performed efficiently on a global scale to quantify the geometric style. Through tuning of the objective weights, the optimized design can have a balance of the optimal structural performance and the desired visual geometric features as determined by the user. Numerical examples are included to demonstrate and validate these methods. Using the current approach, three-dimensional optimization is not implemented, but the methods are similar and will be considered for future work.

The work accomplishes the following:

(a) The geometric result from a topology optimization analysis can be influenced efficiently using a reference design rather than directly copying the reference structures.
(b) The work is an early example using a deep learning model to define objectives and/or constraints for topology optimization, expanding available design objectives and/or constraints.
(c) The weighted objective formulation presents a simple method to add additional constraints to the problem for use with new optimizers developed for machine learning and increased performance.

The paper is organized as follows. In Section 2, the optimization framework with the neural style transfer algorithm, topology optimization formulation, and post-processing filter are described. In Section 3, three cases are presented demonstrating the method with different design problems and reference inputs. In Section 4, the performance of the method and the effects of the neural style transfer parameters and post-processing filter are discussed. Section 5 provides conclusions and future improvements to the method.

## 2. Materials and Methods

**Figure 1:**
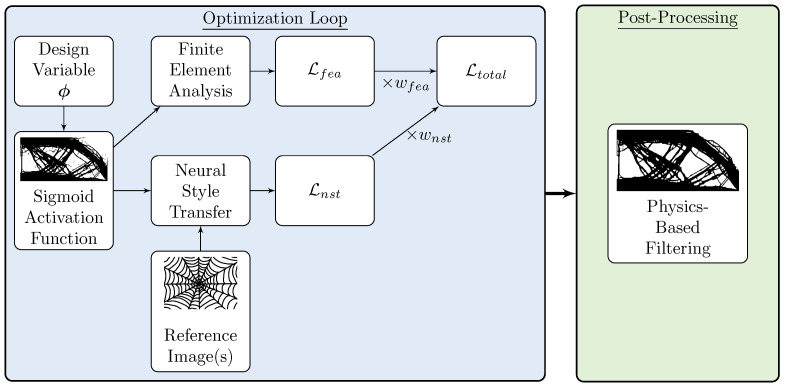
Op-Level Flowchart for each iteration.

This section describes the weighted objective approach for topology optimization developed for this work. Each objective and constraint, including the structural objective and neural style transfer loss, are summed to develop a single loss function to be optimized. Figure 1 illustrates how each iteration of the analysis runs, starting with the design variable, ϕ, and concluding with the total loss sent to the optimizer. The unconstrained design variable is converted with the sigmoid function to be between 0 and 1. The result is used to calculate the loss, L, for the given design problem with the objective function value from finite element analysis and comparing the geometric style with the reference image(s) using neural style transfer through mean squared error (MSE). Both results are multiplied by a weight, *w*, and summed to form the total loss used in the optimizer. After the optimization is complete, the result is post-processed using a physics-based filter to remove extraneous artifacts left from the optimization and smooth the result for manufacturing.

Traditionally, the topology optimization analysis is performed as a single-objective, multi-constraint problem such as:

(1) $$\begin{array}{cc} min & \;f(x)\\ s.t. & \;g_{i}(x)=c_{i},\;for\;i=1,\ldots ,n,\\ & \;h_{j}(x)\;\geq \;d_{j},\;\mathrm{for}\;j=1,\ldots ,m \end{array}$$

where f(x) is the objective function to be optimized (e.g., compliance, mass, etc.), $g_{i}\left(x\right)$ are the equality constraints for *n* equations, $h_{j}\left(x\right)$ are the inequality constraints for *m* equations, and *x* is the design variable constrained between 0 and 1. Although this formulation is suitable for current structural optimizers such as Optimality Criterion or Method of Moving Asymptotes (MMA) [22], optimizers for machine learning problems such as Adam [23] are formulated as a single loss function. Adam was developed to be computationally and memory-efficient for a large number of parameters and use on graphics processing units (GPU). Performing neural network operations on GPUs greatly improves the speed per iteration and reduces the overhead of the neural style transfer calculation [24]. With these considerations and the prevalence of the use of the Adam optimizer, it was chosen for this work.

The loss function is therefore defined as a weighted objective function, where each constraint and objective equation from Equation (1) is considered as a loss term, $L_{i}$, with a corresponding weight, $w_{i}$. The summation of each loss term and weight then forms the function to be optimized, see Equation (2).

(2) $$L_{total}=\sum_{i}w_{i}L_{i}$$

With the machine learning optimizers, the design variables are not constrained between 0 and 1 as seen with the current structural optimizers presented. An activation function is used to convert the design variable, ϕ, to the elemental densities, x. For this work, the sigmoid function is used as it is continuously differentiable, see Equation (3).

(3) $$\mathrm{Sigmoid}\left(\phi \right)=x=\frac{1}{1+\exp(\phi )}$$

**Figure 2:**
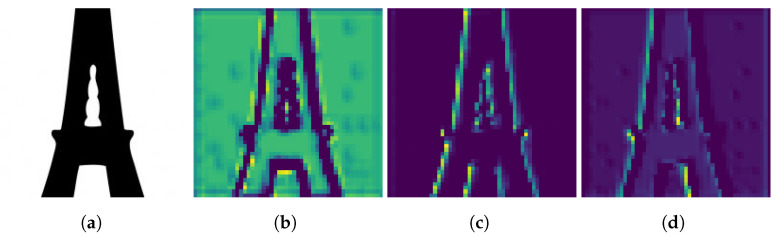
Examples of the activations of selected convolutional filter layers within the VGG-19 neural network for an (**a**) example input. The filters shown exhibit greater activation for (**b**) neighborhood of similar pixels, (**c**) the left outer border of the structure, and (**d**) the right inner border of the structure.

The neural style transfer method is based upon the work by Gatys et al. [21]. The convolutional filters of a pre-trained convolutional neural network are used as local feature extractors for an input. Within the work, the VGG-19 neural network [25] is trained to classify images to one of the over 80,000 sets found in the ImageNet database [26]. The network is modified for use with neural style transfer, using average pooling layers rather than max pooling. With such a variety of images, the convolutional layers are better suited to recognize features on a large variety of inputs. Early convolutional layers in the network (i.e., conv1_1) capture close local features of the input. Deeper layers, through stacked convolutional layers and pooling (i.e., conv5_1), correspondingly extract features from a larger region of the input. The different region scales from the extracted layers smoothly integrate the geometric features to the new design. Figure 2 shows how the neural network can extract the features of an input. This network architecture, also pretrained for classification of the ImageNet database, is also used for this work. The reader is referred to Simonyan et al. [25] and Gatys et al. [21] for the VGG-19 network architecture and modifications for neural style transfer.

Using solely the convolutional layer for comparison between the input to be optimized and the reference, the optimizer would modify the input to be a copy of the reference to best reduce the loss. To determine the style representation, the activation across the entire selected filter must be used for comparison. To achieve this, the Gram matrix, $G_{ij}^{l}$, is calculated as the inner product of the vectorized feature map, $F_{ij}^{l}$, for layer *l*, where $F_{ij}^{l}$ is the activation of the $i^{th}$ filter at position *j* of layer *l*, see Equation (4).

(4) $$G_{ij}^{l}=\sum_{k}F_{ik}^{l}F_{jk}^{l}$$

To calculate the loss function for the style representation for layer *l*, the mean squared error loss between the Gram matrices of the input and style images is used, where $N_{l}$ and $M_{l}$ are the lengths of the Gram matrix of the input to be optimized, $G_{ij}^{l}$, and the Gram matrix of the reference input, $A_{ij}^{l}$, for layer *l*, respectively. This, as well as the derivative for the design sensitivities, are shown in Equations (5) and (6). The convolutional layers conv1_1, conv2_1, conv3_1, conv4_1, and conv5_1 of the VGG-19 network are used to represent the style transfer loss. Liu et al. [27] provides the mathematical representations of the convolutional layers used in the VGG-19 network.

(5) $$L_{l}=\frac{1}{4N_{l}^{2}M_{l}^{2}}\sum_{i,j}{\left(G_{ij}^{l}-A_{ij}^{l}\right)}^{2}$$

(6) $$\frac{dL_{l}}{dF_{ij}^{l}}=\frac{1}{4N_{l}^{2}M_{l}^{2}}{\left({\left(F^{l}\right)}^{T}\left(G^{l}-A^{l}\right)\right)}_{ji}$$

The objective function of the topology optimization analysis for this work is based on the 88 line MATLAB script by Andreassen et al. [28]. The script details an efficient two-dimensional topology optimization problem using a Cartesian mesh. The structural objective presented in the paper is to minimize compliance, or maximize stiffness, for the design and load conditions. This formulation is self-adjoint which simplifies the sensitivity analysis. The equations for compliance and the sensitivities are as follows:

(7) $$\begin{array}{cc} L & =U^{T}KU=\sum_{e=1}^{N}\left(E_{min}+\left(E_{0}-E_{min}\right)x_{e}^{p}\right)u_{e}^{T}k_{0}u_{e} \end{array}$$

(8) $$\begin{array}{cc} KU & =F \end{array}$$

(9) $$\begin{array}{cc} \frac{dL}{dx_{e}} & =-px_{e}^{p-1}\left(E_{0}-E_{min}\right)u_{e}^{T}k_{0}u_{e} \end{array}$$

where x is the input variable vector containing element densities $x_{e}$; K, U, and F are the global stiffness matrix, displacement vector, and force vector, respectively; $u_{e}$ and $k_{0}$ are the element displacement vector and element stiffness matrix, respectively; *p* is a penalty term for the element densities; $E_{0}$ and $E_{min}$ are the maximum and minimum allowable Young’s moduli for solid and void material, respectively; and *N* is the number of elements used for the domain. To avoid checkerboard patterns, a convolutional filter is applied to the sensitivities of the compliance calculation [28]. The convolution is defined as follows:

(10) $$\frac{\hat{dL}}{dx_{e}}=\frac{1}{\max\left(\gamma ,x_{e}\right)\sum_{i\in N_{e}}H_{ei}}\sum_{i\in N_{e}}H_{ei}x_{i}\frac{dL}{dx_{i}}$$

(11) $$H_{ei}=\max\left(0,r_{\min}-\Delta \left(e,i\right)\right)$$

where $N_{e}$ is the set of elements with a center-to-center distance between the current element for the sensitivity, $x_{e}$, and an additional element, $x_{i}$, less than the user defined radius, $r_{\min}$, γ is a small value equivalent to the void density to avoid division by zero, and $H_{ei}$ is the weight factor for the additional element, $x_{i}$.

Two additional constraints were considered with the total loss function to achieve the desired results for the analysis: volume fraction and standard deviation. For the volume fraction, it is defined as the mean absolute error between the current volume of the design, V(x), and the desired volume of the design, $V_{0}$, see Equation (12). Other formulations including the mean squared error were considered but the mean absolute error achieved results closer to the desired volume fraction. The standard deviation term is used to encourage the design to achieve a true 0/1 distribution and is defined as the standard deviation of the element densities. If inputs with intermediate densities are used, this ensures the final design achieves a 0/1 distribution rather than incorporating the intermediate densities from the reference input. This term would be subtracted from the total loss rather than summed, see Equation (13), where x is the input variable vector containing element densities $x_{e}$, μ is the mean of the input vector, and *N* is the number of elements in the input vector.

(12) $$L=|V\left(x\right)-V_{0}|$$

(13) $$L=-\sqrt{\frac{\sum_{i}{\left(x_{i}-\mu \right)}^{2}}{N}}$$

Although topology optimization alone encourages smooth designs to satisfy design constraints, the additional neural style transfer objective with a large weight or poorly suited reference design can introduce objects disconnected or minimally connected to the main design. Martinez et al. and Hu et al. have also presented this issue in their works [7,8]. To overcome the issue, each used an additional constraint within the optimization to discourage the formation of these objects. Martinez et al. suggests adding self-weight to the design problem. However, it is determined the design may not converge properly without relaxation of other constraints [7]. Hu et al. propose two adaptive regulations for the texture appearance weight. The weight would be calculated for each neighborhood of texture, reducing the appearance weight in void regions and avoiding disconnected or minimally connected objects [8]. However, the method described would not be appropriate for this work. The geometric features for this work are calculated on a global scale, not local. Integrating the method would add to the computational cost of each optimization iteration.

To avoid disconnected or minimally connected objects in the final design without great additional computational cost, a post-processing filter is introduced. Image processing techniques such as erosion and dilation were found to remove important load-carrying members or close features introduced from the reference. Similar to Groen and Sigmund [29], a physics-based filter is introduced to remove these disconnected objects without affecting the load-carrying members.

Through experimentation, it was found the equivalent von Mises stress at each element, $\sigma_{e}^{vM}$, in the disconnected objects of the final design was minimal compared to the fully connected objects of the design. The formulation is derived from the stress constrained topology optimization method by Holmberg et al. [30]. Using the result from last iteration of the weighted objective optimization, the equivalent stress for each element was calculated as follows:

(14) $$\begin{array}{cc} \sigma_{e}\left(x_{e}\right) & =EBu_{e} \end{array}$$

(15) $$\begin{array}{cc} \sigma_{e}\left(x_{e}\right) & ={\left(\begin{array}{ccc} \sigma_{xx} & \sigma_{yy} & \tau_{xy} \end{array}\right)}^{T} \end{array}$$

(16) $$\begin{array}{cc} \sigma_{e}^{vM}\left(x_{e}\right) & ={\left(\sigma_{xx}^{2}+\sigma_{yy}^{2}-\sigma_{xx}\sigma_{yy}+3\tau_{xy}^{2}\right)}^{\frac{1}{2}} \end{array}$$

where E is the constitutive matrix, B is the strain-displacement matrix, $u_{e}$ is the element displacement vector for element $x_{e}$ found from Equation (8), $\sigma_{e}\left(x_{e}\right)$ is the two-dimensional stress tensor with components for a Cartesian coordinate system, and $\sigma_{e}^{vM}$ is the equivalent von Mises stress.

From the analysis, the elements with an equivalent stress value below a threshold would be set as void material. For the numerical examples presented in this work, it was found a threshold of 10% of the elements with the least stress produced favorable results. Examples demonstrating the effectiveness of the filter are presented in Section 4.

## 3. Numerical Examples

**Figure 3:**
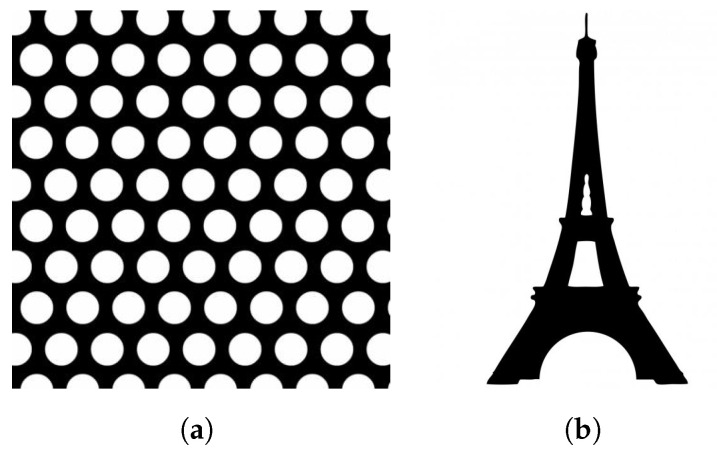
Reference design inputs for examples include (**a**) circular mesh and (**b**) Eiffel tower.

In this section, multiple two-dimensional examples are presented to show the capabilities of the proposed work. The examples were created using a Python script built around the PyTorch machine learning library implementing the method. Table 1 details the parameters and design domain used for each of the examples. The design problems presented include the MBB-beam and the cantilever beam, where black and white represent solid and void material, respectively. Both problems use the same values, as the values produce quality results for the examples and simplify the problems for the reader to reproduce the presented results. Figure 3 shows the reference inputs used for the examples.

### 3.1. MBB Beam

**Figure 4:**
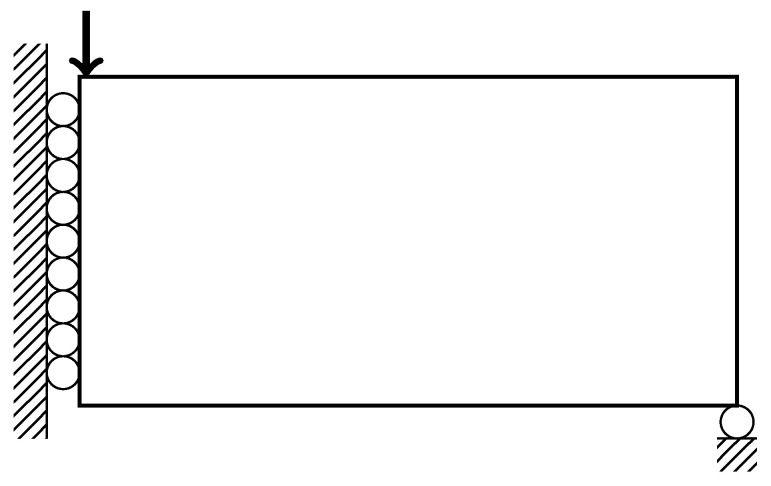
Conditions for MBB Beam.

**Figure 5:**
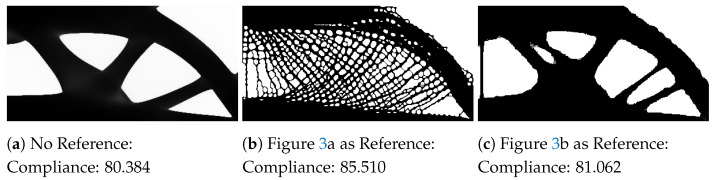
Examples of the MBB beam examples with the compliance values for each design.

The MBB beam is a common design problem among topology optimization research as a benchmark for new methods [28]. The design problem is illustrated in Figure 4. The left edge of the beam has zero horizontal displacement, and the bottom right node has zero vertical displacement. The force is applied to the top-left node. Using the parameters described in Table 1, the baseline design and designs influenced by the style input are shown in Figure 5.

Figure 5a shows the optimized design for the MBB design problem without any reference input. The optimized design is characterized by three large supporting members inside the design envelope to support the force.

Observing the reference for the result shown in Figure 5b, the reference input is composed of a repeating circular mesh structure. Through the neural style transfer objective, the corresponding mesh is applied to the inner structure of the design, replacing the three supporting members found in Figure 5a. Although the size of mesh beams more closely reflect the reference image, the directions of the beams closely follow the standard result members to satisfy the compliance. This compromise results in irregular holes, rather than circular, in the final design. This may not fully achieve the desired geometric features, but the beams within the mesh better align with the ideal direction to support the load with the influence of the many holes from the reference input. The outer envelope of the design better matches the standard result, not incorporating the mesh from the reference input. As the outer envelope has larger members, it is determined the region greatly contributes to the structural performance. The optimizer converged to a solid region for improved structural performance, rather than including the holes from the reference design. When increasing the weight of the neural style transfer loss, a greater portion of the design incorporates the mesh, ultimately encompassing the full design space. Although this would satisfy the ideal geometric style, the structural performance is greatly diminished.

Figure 5c uses a reference input composed of a tower. The input is symmetrical but does not have many repeating elements as found for Figure 5b. The beams found in the reference are slender, with some material removed as it converges near the top of the tower. In the optimized design, the outer envelope is similar to the standard result. However, five supporting members are used inside the design envelope, rather than three found in the standard result. The voids are rounder to match the smooth curves of the reference and incorporate a hole in the leftmost member which is also found in the reference.

Reviewing all three designs reveals common elements among them, notably the outer envelope and the directions of the inner members. Even with the different reference inputs, these elements were considered crucial to maintaining the structural performance of the final design. Figure 5b heavily applies the mesh to the inner members to satisfy the neural style transfer objective. The reference input for Figure 5c is comparably simpler and the optimized result is nearly identical to the standard result except for the additional inner members. Although there are design differences between all three results, the structural performances of both stylized results are still maintained within 10% of the baseline design.

### 3.2. Cantilever Beam

**Figure 6:**
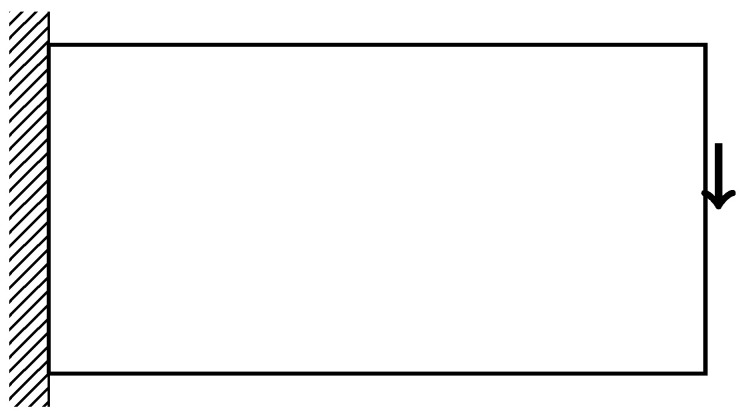
Conditions for Cantilever Beam.

**Figure 7:**
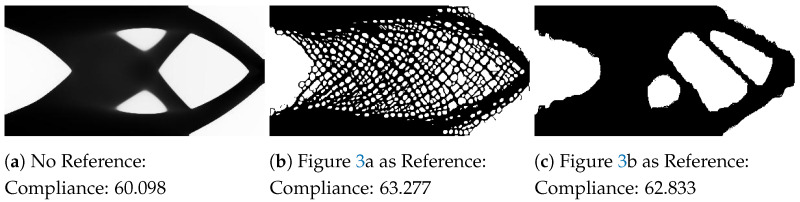
Examples of the cantilever beam examples with the compliance value for each design.

The cantilever beam design is inspired by the work by Wu et al. [10] for infill optimization. The design problem is illustrated in Figure 6. The left edge of the beam is fixed in all directions. A downward vertical force is applied to the middle of the right edge of the beam. Using the parameters described in Table 1, the baseline design and designs influenced by the style input for this design problem are shown in Figure 7.

Figure 7a shows the optimized design with no reference input. Although the problem is not symmetrical, the optimized design is. Two small members inside the outer envelope provide additional rigidity to improve the structural performance. For this example, the reference inputs used for the MBB beam designs are used here.

Figure 7b uses the circular mesh pattern as the reference input. As seen in Figure 5b, the outer envelope of the design is very similar to the standard result. In this design, the mesh is incorporated at the edges of the envelope to satisfy the neural style transfer objective. To further reduce the neural style transfer loss, much of the interior structure is composed of circular mesh elements. The beams follow the directions of the two small members in the standard result and are tightly packed to resemble the reference input.

Figure 7c follows the simple design of the standard result but incorporates more features from the reference compared with Figure 5c. The interior members are correspondingly thinner and are present in only one direction to support the asymmetric load. The void areas also have round edges compared with the sharper corners in Figure 7a.

### 3.3. Using Multiple Reference Designs

**Figure 8:**
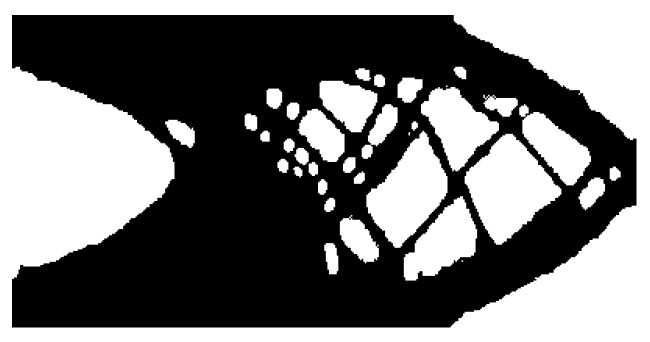
Cantilever beam design using both reference inputs from Figure 3; Compliance: 61.147.

A benefit of this method is multiple reference designs can be utilized for the neural style transfer and balance the geometric features of multiple sources. The additional reference is added as another objective for the multi-objective formulation. Figure 8 shows the results for two inputs.

Performing the optimization did not impact the performance of the optimizer. The average amount of time for each iteration was 1.8765 s. As presented in Table 2, this is comparable between two common optimizers used for TO solving similar design problems without the neural style transfer: Optimality Criterion and MMA [22]. The reason for this is that the gram matrices for each reference are stored and not recalculated for each iteration. The mean squared error calculation is a relatively fast operation and does not impact the optimization speed.

The result in Figure 8 does show resemblance to both provided references. The smooth edges of the tower are present at the solid-void boundaries in the optimized design. Smaller members are used as well as found in Figure 7c. To satisfy the circular hole pattern, many small holes were added to the members as found in Figure 7b but are not as prevalent as they would deviate from the tower reference input in Figure 7c.

## 4. Discussion

### 4.1. Performance

**Figure 9:**
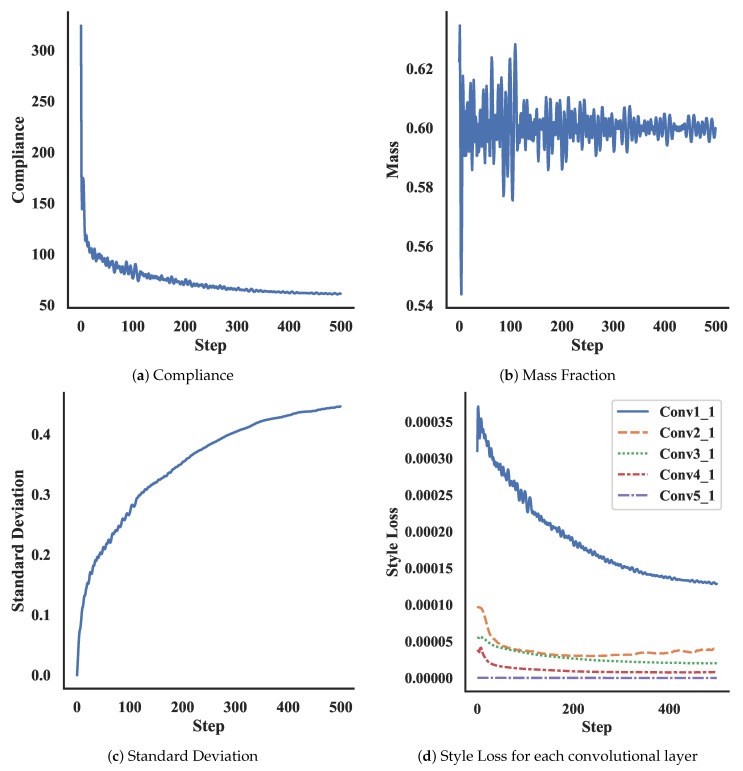
Optimization convergence plots for result in Figure 7c.

Figure 9 shows the convergence history for the result shown in Figure 7c for each function: compliance, mass fraction, standard deviation, and style loss for each layer. All presented results follow similar histories. At the start of training, the optimizer greatly improves the compliance value and style losses and exceeds the desired volume fraction. After some steps, more material is removed and the volume fraction correspondingly decreases until it falls below the desired volume fraction, with improving compliance and style loss values. Towards the later stages, the improvements to the compliance and style loss diminish. For each oscillation of the volume fraction, the compliance and style loss values correspondingly oscillate. The values continue to improve at a much slower rate until the given number of steps is reached. Although the losses have not converged after the training procedures, the overall structure changed minimally at the end of the optimization, only varying in particular regions. These regions were processed using the physics-based filter to complete the optimization. With different parameters from Table 1, more or less iterations may be required to achieve a final design.

The results presented in this work are characterized as deterministic, and therefore do not require a statistical analysis. The CNN used for neural style transfer is pre-trained and is not updated between results. The initialization parameters, shown in Table 1, are also identical for repeated results, including the initial density of the design space. As the initial state for a set of parameters is always identical, the gradients for descent are also equal and the optimizer follows identical convergence paths for repeated analyses.

Table 2 shows the comparison of the Optimality Criterion optimizer and the MMA optimizer [22] using the MATLAB implementation of the 88 line topology optimization script by Andreassen et al. [28] with the Python implementation of this work using the Adam optimizer [23] with and without the neural style transfer constraint. This was performed with an 8-core Intel Xeon 3.7GHz processor, 128 GB RAM, and an NVIDIA Quadro RTX 6000 GPU.

Using the Adam optimizer with the given equipment is on par with the linear Optimality Criterion optimizer and much faster than the MMA algorithm. Although it is faster, more iterations are necessary to ensure a good result. It would be beneficial to use techniques for machine learning to speed up the accuracy of the network for this. One such example includes transfer learning from a coarse result. The design could be optimized for a low-resolution domain. The design would then be scaled to the finer resolution to complete the optimization in fewer steps. Another example would be learning rate annealing. High learning rate values result in large improvements early in the optimization process. After many steps, the high learning rate starts to overshoot and the accuracy fails to improve further. Learning rate annealing would reduce the learning rate for this situation. The smaller learning rate would help perform smaller steps to better achieve the minimum. A small learning rate could be used at the beginning of the optimization, but many more steps would be required compared with the annealed method.

### 4.2. Post-Processing

Without the neural style transfer objective, the optimized design is smooth and continuous with no artifacts left from the optimization. The neural style transfer objective, however, acknowledges the full design domain to optimize the geometric style. If a large area of the design does not contribute to the overall structural performance, the optimizer could satisfy the neural style transfer objective by adding material with the geometric features of the reference.

**Figure 10:**
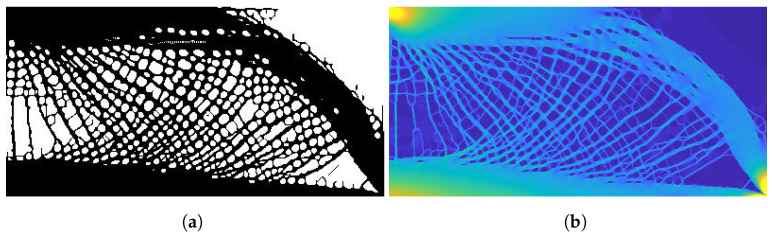
Effects of the stress-based post-processing filter (**a**) before and (**b**) the equivalent von Mises stress result. The post-processed result is found in Figure 5b.

Figure 10a shows the result from Figure 5b immediately after optimization. The outer envelope contains many small members minimally connected to the design but still following the circular reference design. In the void material near the bottom right support, a disconnected member is also present. Traditional image processing techniques would not work for this design, as a filter that would remove the artifacts would also impact the desired circular mesh, introducing more minimally connected objects.

It was then found the minimally connected objects in both areas experience an equivalent von Mises stress, similar to the void material areas surrounding them. Figure 10b shows the equivalent von Mises stress for the result in Figure 10a. Although the disconnected members are visible in the structural result, the members exhibit very low stress and are indistinguishable from the void stress values. Using the threshold method described in Section 2, the elements with stress values below the threshold, including the disconnected members, are set to void material and removed from the final result.

### 4.3. Connectivity

As seen in Figure 10, some structures are disconnected from the main structure after the optimization analysis. The post-processing filter can remove these objects effectively. However, future work should be done to limit these artifacts during the optimization.

Such improvement could come from the addition of a stress constraint to the weighted objective function. The effectiveness of the post-processing filter shows the objective would reduce the number of the minimally connected objects. As discussed in Section 2, the calculation of the stress would impact the performance. The efficiency of the calculation would have to be considered during the implementation.

**Figure 11:**
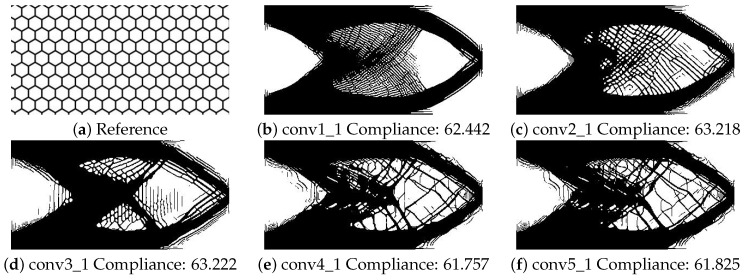
Examples of the cantilever beam using different layers of the neural network.

**Figure 12:**
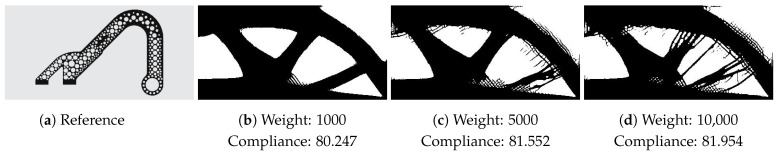
Examples of the MBB beam using different weights for the neural style transfer.

Through repeated training procedures, the weights of the individual style layers would be adjusted rather than using a single weight applied to all layers to improve the result. As shown in Figure 11 and Figure 12, each layer contributes a different aspect of the reference input to the design, and adjusting the layer weights would change the final result. The images in the figures were not post-processed with the stress-based filter to show the result after optimization. Using conv1_1, it is understood a mesh would satisfy the reference input. Using conv2_1 and conv3_1, the thickness of the members is found. Using conv4_1 and conv5_1, the ideal angles of the members emerge in the design. Through the use of the system, the results and parameters of satisfactory designs would be saved to a database. Through searching or training another machine learning network of the database, parameter weights for each layer would be suggested to achieve ideal results.

Additionally, this work uses the original neural style transfer formulation by Gatys et al. [21]. As described in Jing et al. [31], newer implementations are in development to improve the results. Jing summarizes the extensions to the original approach and various loss functions of the layer activations to improve the results. Adjusting the neural style transfer to one of the described methods would require additional investigation for further research.

## 5. Conclusions

In this work, a novel approach to generate topology optimized structures is proposed using a pre-trained neural network to quantify the desired geometric style of the optimized design. The conclusions drawn are as follows:

(a) The neural style transfer quantifies the geometric features of the reference and optimized designs efficiently using a Gram matrix calculation of the pre-trained convolutional filter activations for a neural network classifier. As such, the features of the input are replicated rather than directly copied in the optimized design, which expands the number of applicable inputs.
(b) The weighted objective formulation presents a simple method to add additional constraints to the problem and tune the influence for each constraint. The formulation also allows the utilization of new optimizers developed for machine learning and increases performance.

Although compliance is used for the objective, it is possible to use other structural objectives such as natural frequency or stress minimization using the same optimizer. Performing these analyses would further validate and improve the usefulness of the proposed method.

Additionally, recent neural style transfer methods have been developed which improve upon the original formulation used in this work; see Jing et al. [31] for a comprehensive review. Berger and Memisevic translate the feature maps to better capture spatial and symmetric arrangements [32]. A recent work by Gatys et al. allows for spatial control, limiting the style transfer to regions of the design and not the global structure [33]. Using these methods could improve the final results of the optimization process, eliminating the need for the stress-based post-processing filter. Similarly, the optimization need not be done as a linear summation of all objectives. Prioritized optimization is an example of an alternative method [34]. Using this method, multiple optimal solutions of one objective function are used to find equally optimal solutions for an additional objective function. Rather than using weights as found in the current linear summation, the method will iterate through solutions that limit the error for each objective function. These improvements will be left as future work for the authors.

Although topology optimization is most useful for three-dimensional CAD design, the neural style transfer implementation used is limited to two-dimensional input. Using three-dimensional voxelized inputs or other deep geometric learning methods, a three-dimensional CNN classifier for CAD files with a similar architecture compared with the network used in this work can be used for neural style transfer. The implementation described by Gatys et al. [21] can be replicated for three-dimensional CNNs. This method is under investigation by the authors.

## Figures and Tables

**Table 1:** Variable Initialization.

Variable	Value
Elements in x-direction	400
Elements in y-direction	200
Filter Radius for Sensitivity Analysis	1.5 elements
Mass Penalty for Finite Element Analysis	3.0
Young’s Modulus	$10^{-6}$–1.0
Poisson’s Ratio	0.3
Force	1.0
Structural Compliance Weight	1
Neural Style Transfer Weight	$10^{3}$–$10^{5}$
Volume Fraction Weight	0.1
Standard Deviation Weight	0.1
Number of Iterations	500
Step Size for Adam Optimizer	0.08

**Table 2:** Average Time per Iteration of Optimizers.

Optimizer	Time (s)
Optimality Criterion (Top88 Formulation) [28]	1.1208
Method of Moving Asymptotes without Neural Style Transfer [22]	2.8790
Adam without Neural Style Transfer	1.0360
Adam with Neural Style Transfer	1.8965

## Data Availability

The data presented in this study are available in the Supplementary Material.

## Supplementary Materials

The following are available online at https://www.mdpi.com/article/10.3390/ma14164551/s1: Source Code.
