# Supplementary figures and images for: Integrating Geometric Data into Topology Optimization via Neural Style Transfer
Source: Materials (Basel). 2021 Aug 13;14(16):4551. doi: 10.3390/ma14164551 (PMC8400862; doi:10.3390/ma14164551)

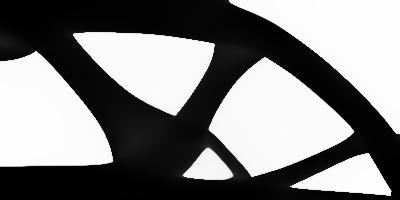

Supplement: Supplementary file 1 [file materials-14-04551-s001.zip › lst-master-Code/Code/Conv_Loss/Transfer0_93_147.jpg]

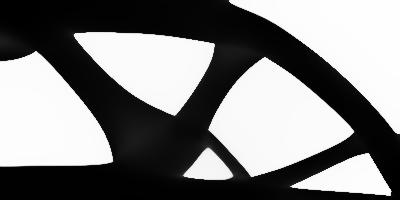

Supplement: Supplementary file 1 [file materials-14-04551-s001.zip › lst-master-Code/Code/Conv_Loss/Transfer1_93_183.jpg]

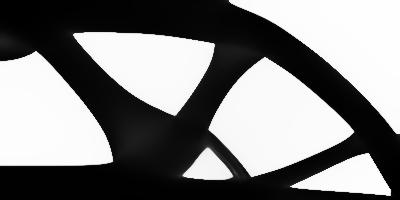

Supplement: Supplementary file 1 [file materials-14-04551-s001.zip › lst-master-Code/Code/Conv_Loss/Transfer2_93_187.jpg]

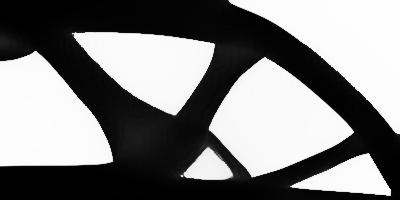

Supplement: Supplementary file 1 [file materials-14-04551-s001.zip › lst-master-Code/Code/Conv_Loss/Transfer3_93_223.jpg]

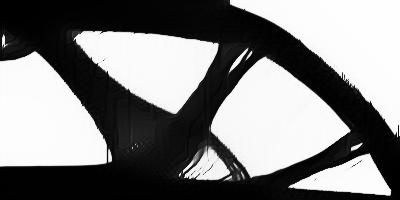

Supplement: Supplementary file 1 [file materials-14-04551-s001.zip › lst-master-Code/Code/Conv_Loss/Transfer4_93_983.jpg]

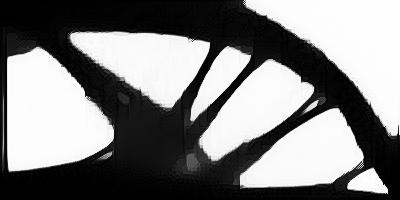

Supplement: Supplementary file 1 [file materials-14-04551-s001.zip › lst-master-Code/Code/Conv_Loss/Transfer5_92_930.jpg]

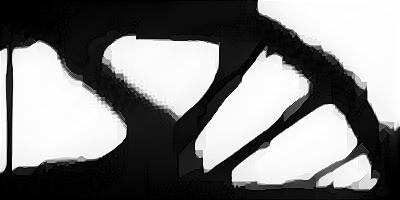

Supplement: Supplementary file 1 [file materials-14-04551-s001.zip › lst-master-Code/Code/Conv_Loss/Transfer6_97_312.jpg]

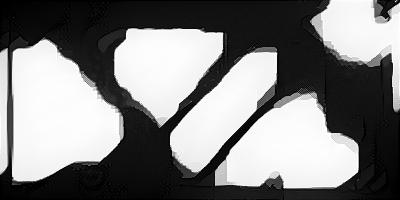

Supplement: Supplementary file 1 [file materials-14-04551-s001.zip › lst-master-Code/Code/Conv_Loss/Transfer7_121_758.jpg]

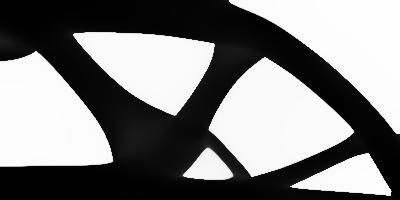

Supplement: Supplementary file 1 [file materials-14-04551-s001.zip › lst-master-Code/Code/Conv_Loss/Transfer_93_147.jpg]

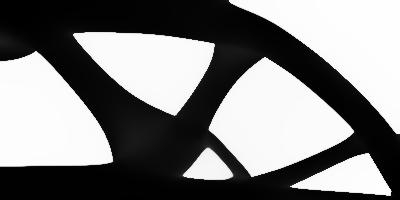

Supplement: Supplementary file 1 [file materials-14-04551-s001.zip › lst-master-Code/Code/Relu_Loss/Transfer0_93_148.jpg]

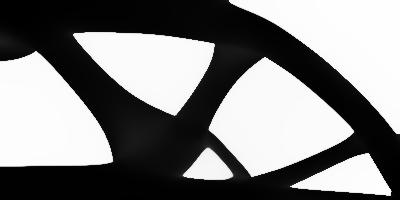

Supplement: Supplementary file 1 [file materials-14-04551-s001.zip › lst-master-Code/Code/Relu_Loss/Transfer1_93_148.jpg]

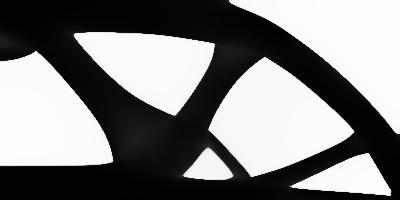

Supplement: Supplementary file 1 [file materials-14-04551-s001.zip › lst-master-Code/Code/Relu_Loss/Transfer2_93_093.jpg]

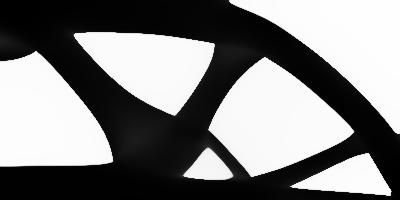

Supplement: Supplementary file 1 [file materials-14-04551-s001.zip › lst-master-Code/Code/Relu_Loss/Transfer3_93_128.jpg]

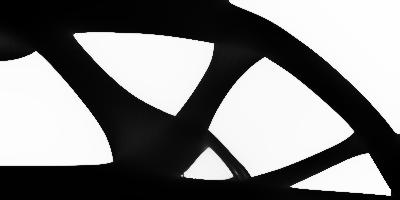

Supplement: Supplementary file 1 [file materials-14-04551-s001.zip › lst-master-Code/Code/Relu_Loss/Transfer4_93_064.jpg]

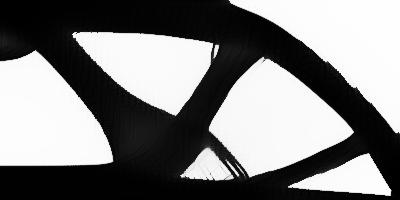

Supplement: Supplementary file 1 [file materials-14-04551-s001.zip › lst-master-Code/Code/Relu_Loss/Transfer5_93_444.jpg]

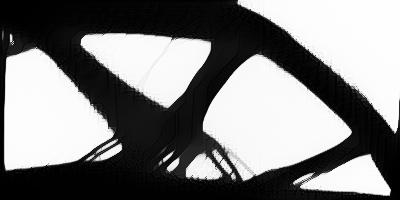

Supplement: Supplementary file 1 [file materials-14-04551-s001.zip › lst-master-Code/Code/Relu_Loss/Transfer6_93_911.jpg]

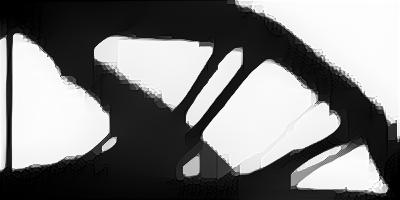

Supplement: Supplementary file 1 [file materials-14-04551-s001.zip › lst-master-Code/Code/Relu_Loss/Transfer7_99_125.jpg]

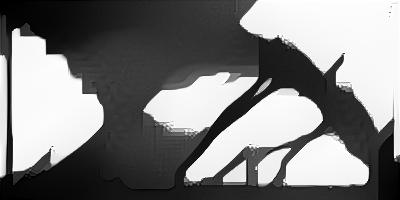

Supplement: Supplementary file 1 [file materials-14-04551-s001.zip › lst-master-Code/Code/Relu_Loss/Transfer8_119_491.jpg]

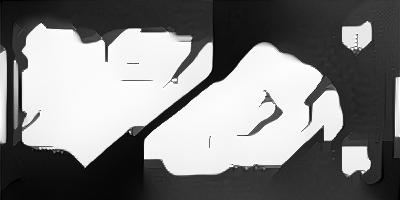

Supplement: Supplementary file 1 [file materials-14-04551-s001.zip › lst-master-Code/Code/Relu_Loss/Transfer9_193_134.jpg]

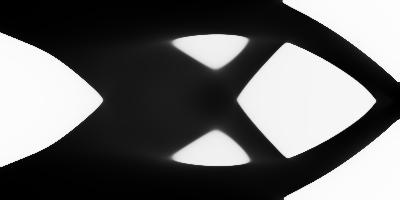

Supplement: Supplementary file 1 [file materials-14-04551-s001.zip › lst-master-Code/Code/Results/Cantilever/Baseline/result.jpg]

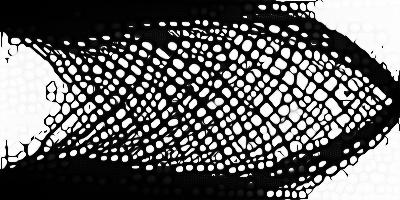

Supplement: Supplementary file 1 [file materials-14-04551-s001.zip › lst-master-Code/Code/Results/Cantilever/circle/result.jpg]

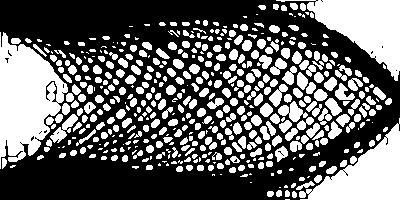

Supplement: Supplementary file 1 [file materials-14-04551-s001.zip › lst-master-Code/Code/Results/Cantilever/circle/round.jpg]

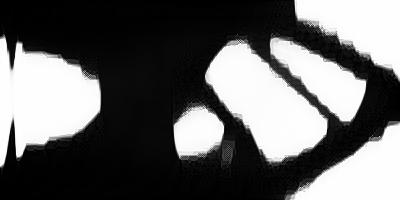

Supplement: Supplementary file 1 [file materials-14-04551-s001.zip › lst-master-Code/Code/Results/Cantilever/tower/result.jpg]

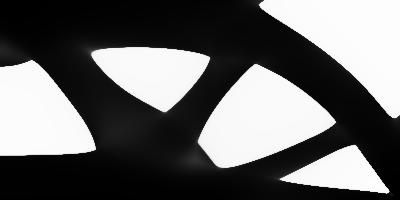

Supplement: Supplementary file 1 [file materials-14-04551-s001.zip › lst-master-Code/Code/Results/MBB/Baseline/result.jpg]

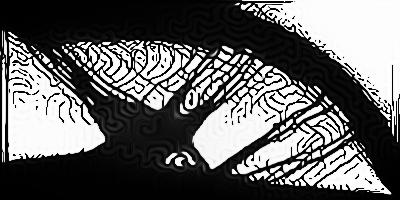

Supplement: Supplementary file 1 [file materials-14-04551-s001.zip › lst-master-Code/Code/Results/MBB/Gosper/result.jpg]

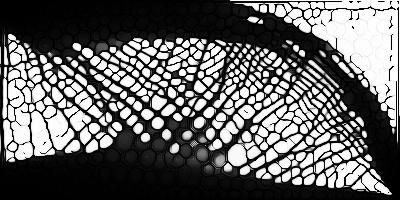

Supplement: Supplementary file 1 [file materials-14-04551-s001.zip › lst-master-Code/Code/Results/MBB/Honeycomb/result.jpg]

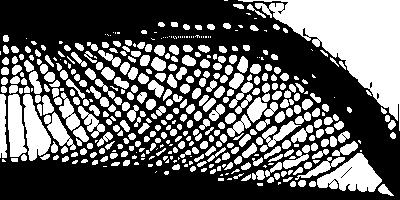

Supplement: Supplementary file 1 [file materials-14-04551-s001.zip › lst-master-Code/Code/Results/MBB/circle/result.jpg]

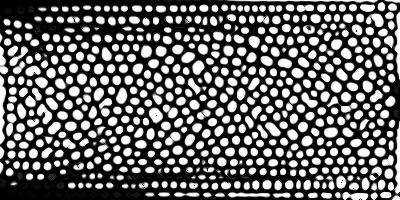

Supplement: Supplementary file 1 [file materials-14-04551-s001.zip › lst-master-Code/Code/Results/MBB/result.jpg]

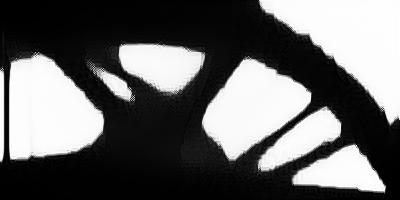

Supplement: Supplementary file 1 [file materials-14-04551-s001.zip › lst-master-Code/Code/Results/MBB/tower/result.jpg]

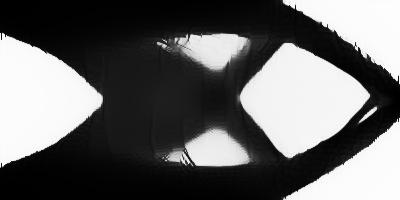

Supplement: Supplementary file 1 [file materials-14-04551-s001.zip › lst-master-Code/Code/Results/empty/10000.jpg]

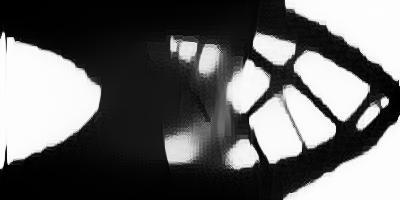

Supplement: Supplementary file 1 [file materials-14-04551-s001.zip › lst-master-Code/Code/Results/empty/100000.jpg]

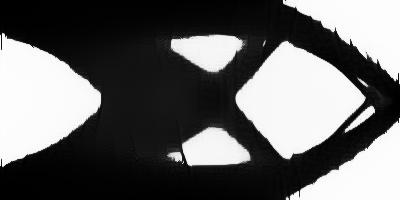

Supplement: Supplementary file 1 [file materials-14-04551-s001.zip › lst-master-Code/Code/Results/empty/20000.jpg]

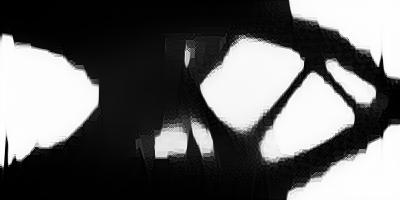

Supplement: Supplementary file 1 [file materials-14-04551-s001.zip › lst-master-Code/Code/Results/empty/200000.jpg]

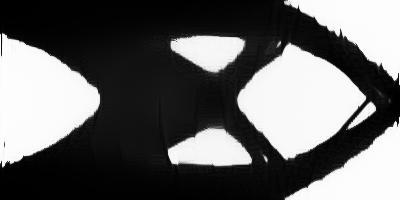

Supplement: Supplementary file 1 [file materials-14-04551-s001.zip › lst-master-Code/Code/Results/empty/30000.jpg]

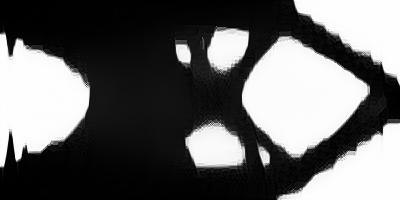

Supplement: Supplementary file 1 [file materials-14-04551-s001.zip › lst-master-Code/Code/Results/empty/300000.jpg]

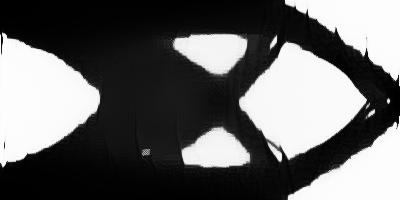

Supplement: Supplementary file 1 [file materials-14-04551-s001.zip › lst-master-Code/Code/Results/empty/40000.jpg]

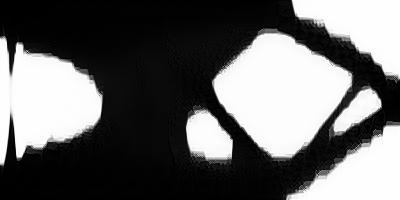

Supplement: Supplementary file 1 [file materials-14-04551-s001.zip › lst-master-Code/Code/Results/empty/400000.jpg]

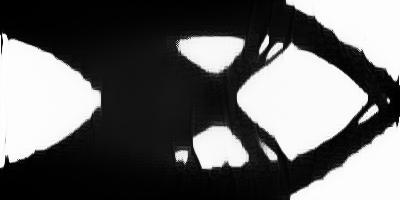

Supplement: Supplementary file 1 [file materials-14-04551-s001.zip › lst-master-Code/Code/Results/empty/50000.jpg]

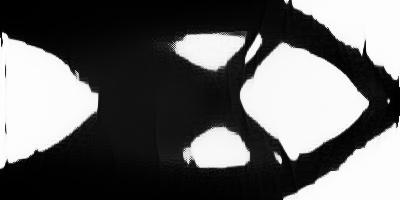

Supplement: Supplementary file 1 [file materials-14-04551-s001.zip › lst-master-Code/Code/Results/empty/60000.jpg]

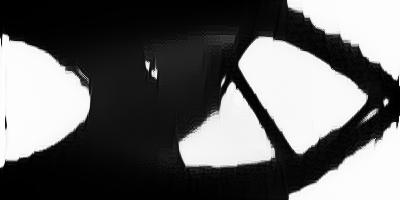

Supplement: Supplementary file 1 [file materials-14-04551-s001.zip › lst-master-Code/Code/Results/empty/70000.jpg]

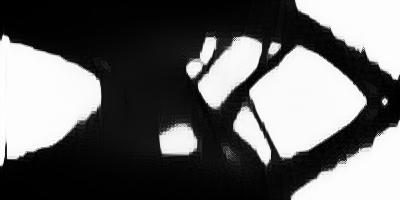

Supplement: Supplementary file 1 [file materials-14-04551-s001.zip › lst-master-Code/Code/Results/empty/80000.jpg]

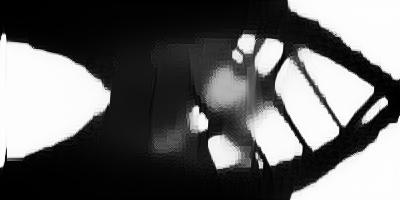

Supplement: Supplementary file 1 [file materials-14-04551-s001.zip › lst-master-Code/Code/Results/empty/90000.jpg]

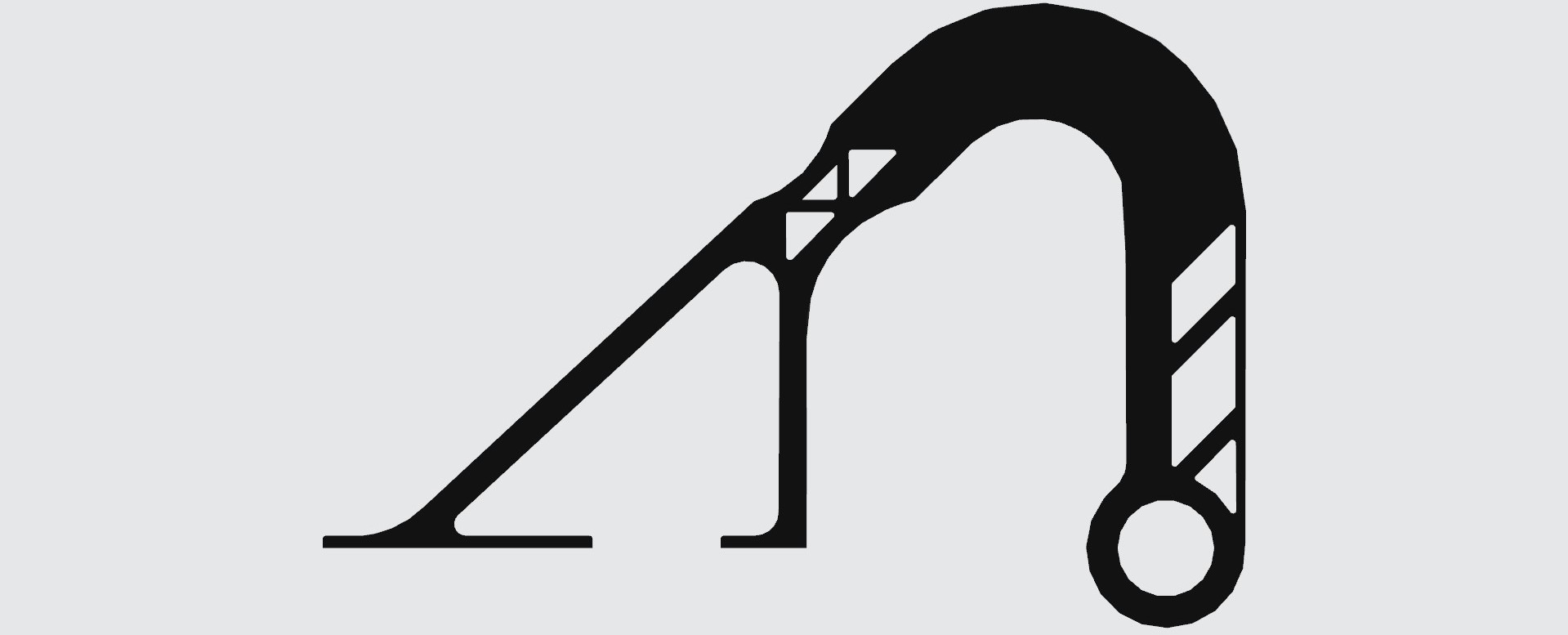

Supplement: Supplementary file 1 [file materials-14-04551-s001.zip › lst-master-Code/Code/ref_images/2P5Bracket.jpg]

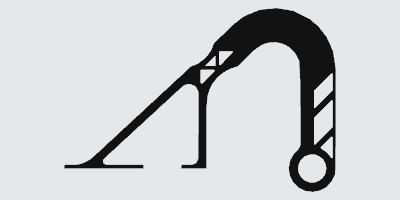

Supplement: Supplementary file 1 [file materials-14-04551-s001.zip › lst-master-Code/Code/ref_images/2P5Bracket_TOPOP3.png]

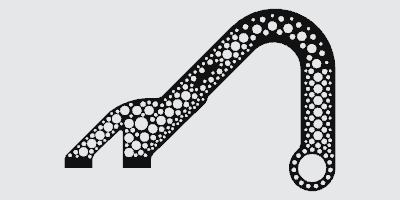

Supplement: Supplementary file 1 [file materials-14-04551-s001.zip › lst-master-Code/Code/ref_images/M_Criado.jpg]

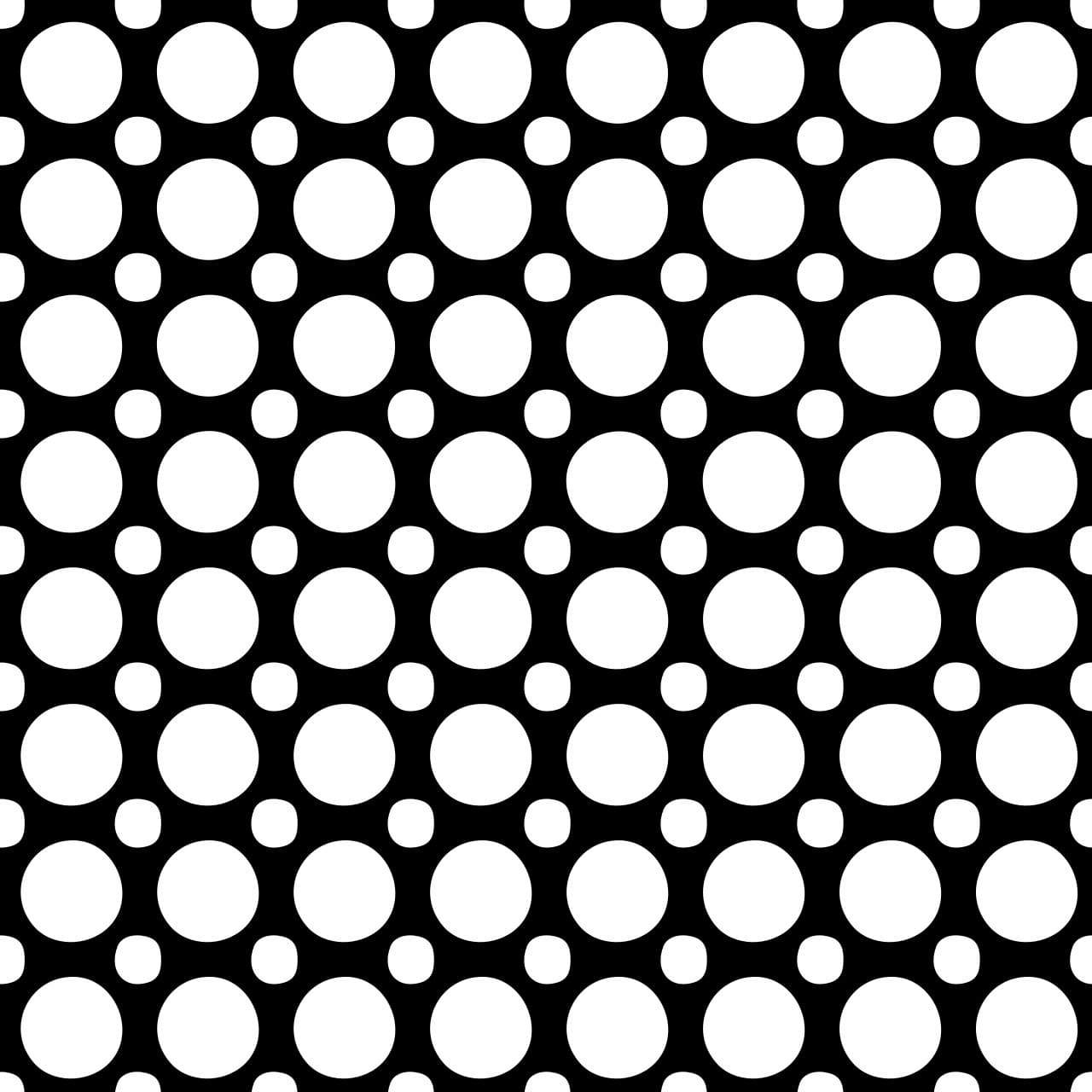

Supplement: Supplementary file 1 [file materials-14-04551-s001.zip › lst-master-Code/Code/ref_images/circle.jpg]

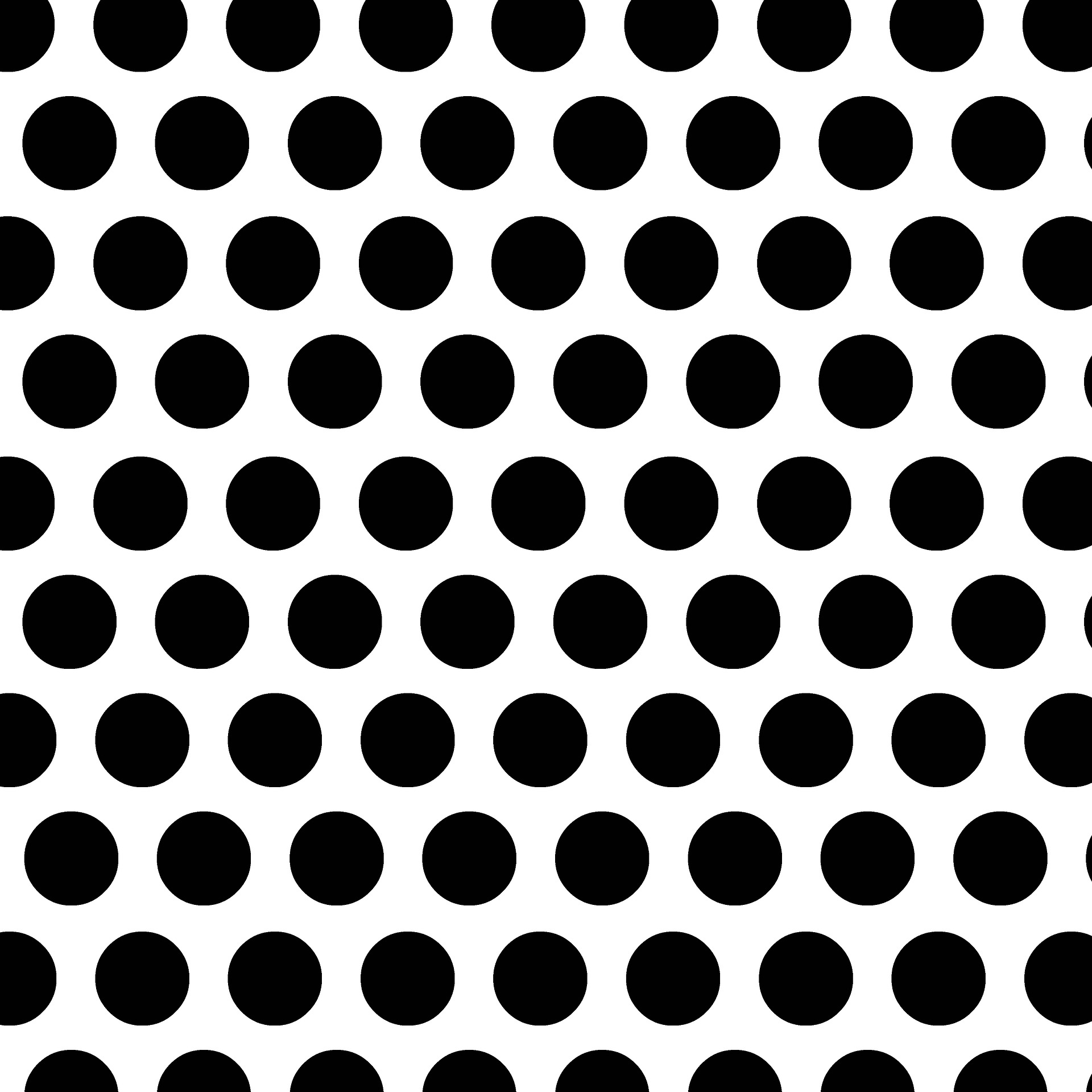

Supplement: Supplementary file 1 [file materials-14-04551-s001.zip › lst-master-Code/Code/ref_images/circle2.jpg]

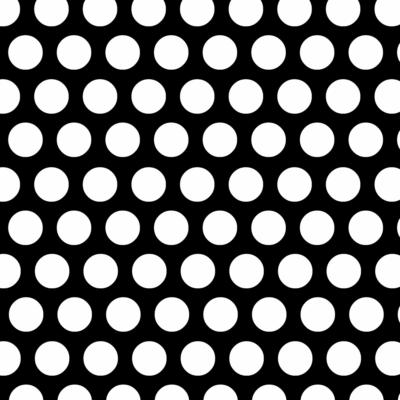

Supplement: Supplementary file 1 [file materials-14-04551-s001.zip › lst-master-Code/Code/ref_images/circle3.jpg]

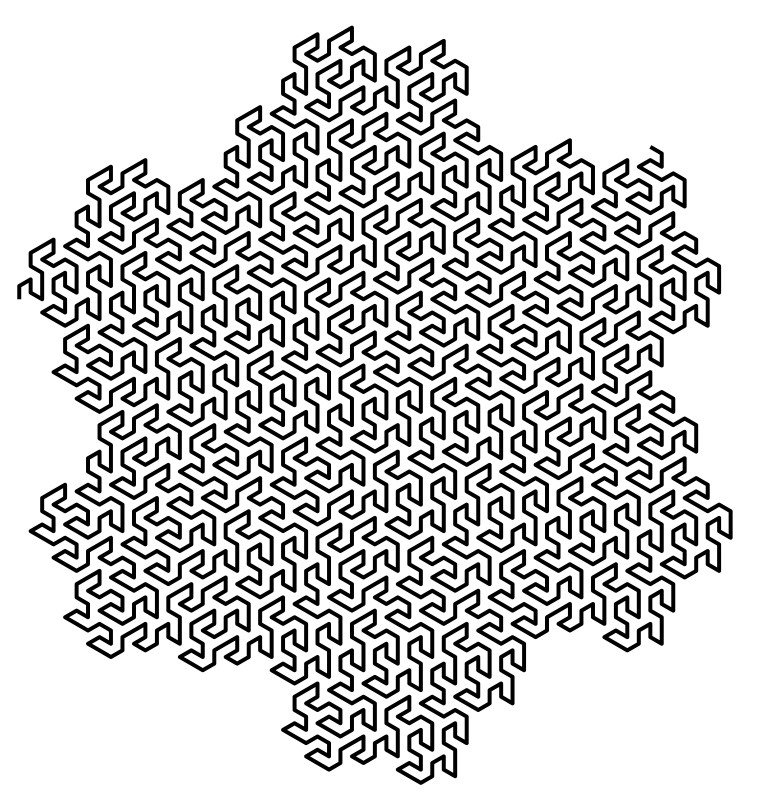

Supplement: Supplementary file 1 [file materials-14-04551-s001.zip › lst-master-Code/Code/ref_images/gosper.jpg]

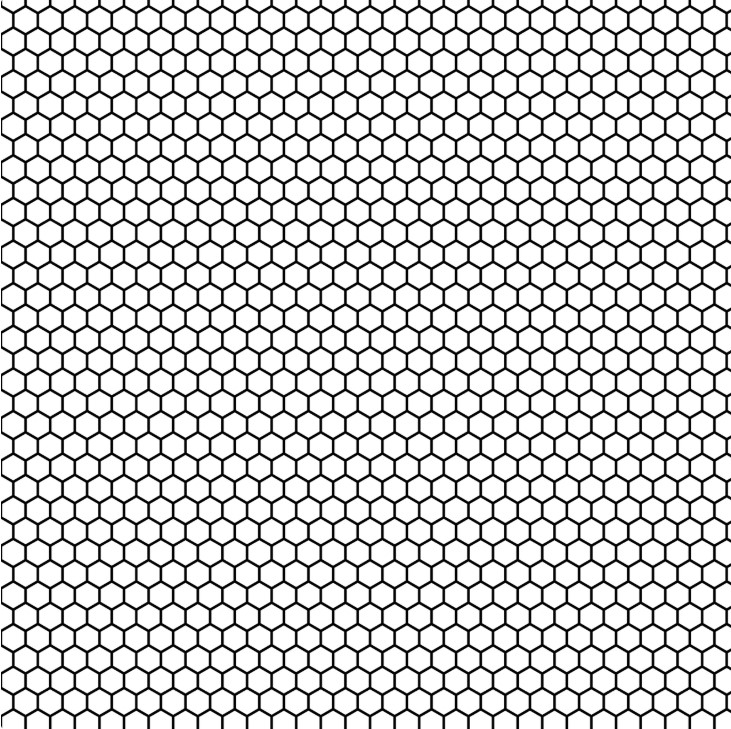

Supplement: Supplementary file 1 [file materials-14-04551-s001.zip › lst-master-Code/Code/ref_images/honeycomb.jpg]

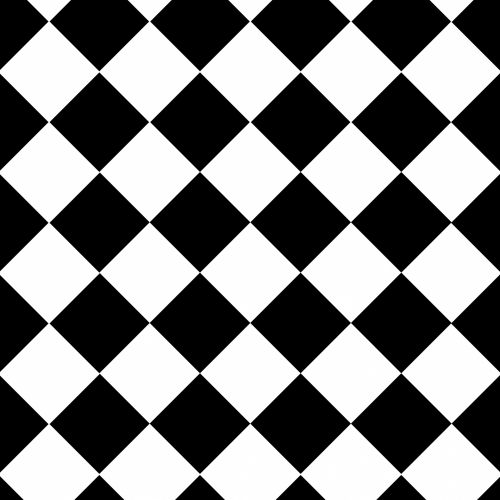

Supplement: Supplementary file 1 [file materials-14-04551-s001.zip › lst-master-Code/Code/ref_images/tile.jpg]

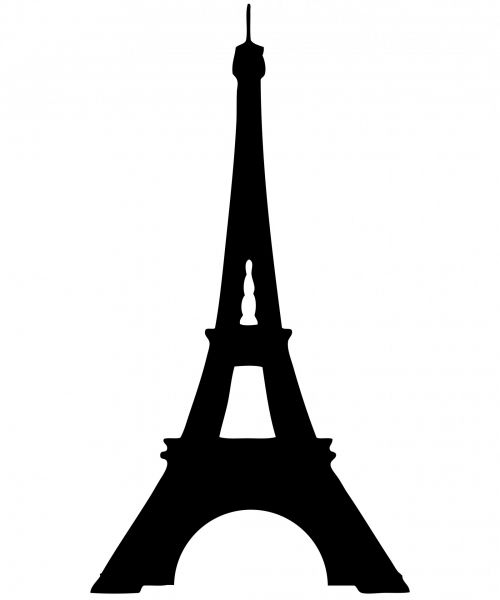

Supplement: Supplementary file 1 [file materials-14-04551-s001.zip › lst-master-Code/Code/ref_images/tower.jpg]
